# Supplementary material for: Comparing the effectiveness and cost-effectiveness of self-management interventions in four high-priority chronic conditions in Europe (COMPAR-EU): a research protocol
Source: BMJ Open. 2020 Jan 19;10(1):e034680. doi: 10.1136/bmjopen-2019-034680 (PMC7044921; doi:10.1136/bmjopen-2019-034680)
Supplement: Author's manuscript [file bmjopen-2019-034680.draft_revisions.pdf]

BMJ Open

BMJ Open is committed to open peer review. As part of this commitment we make the peer review history of every article we publish publicly available.

When an article is published we post the peer reviewers' comments and the authors' responses online. We also post the versions of the paper that were used during peer review. These are the versions that the peer review comments apply to.

The versions of the paper that follow are the versions that were submitted during the peer review process. They are not the versions of record or the final published versions. They should not be cited or distributed as the published version of this manuscript.

BMJ Open is an open access journal and the full, final, typeset and author-corrected version of record of the manuscript is available on our site with no access controls, subscription charges or pay-per-view fees (http://bmjopen.bmj.com).

If you have any questions on BMJ Open's open peer review process please email info.bmjopen@bmj.com

BMJ Open

# Comparing the effectiveness and cost-effectiveness of self-management interventions in four high-priority chronic conditions in Europe (COMPAR-EU): a research protocol



| | |
|---|---|
| Journal: | *BMJ Open* |
| Manuscript ID | bmjopen-2019-034680 |
| Article Type: | Protocol |
| Date Submitted by the Author: | 01-Oct-2019 |





| | |
|---|---|
| Complete List of Authors: | Ballester, Marta; Fundacion Avedis Donabedian, Avedis Donabedian Research Institute (FAD). Universitat Autònoma de Barccelona (UAB); REDISSEC<br>Orrego, Carola; Fundacion Avedis Donabedian, Avedis Donabedian Research Institute (FAD). Universitat Autònoma de Barccelona (UAB); REDISSEC<br>Heijmans, Monique; Netherlands Institute for Health Services Research, (NIVEL)<br>Alonso-Coello, Pablo; Iberoamerican Cochrane Centre, CIBERESP-IIB Sant Pau,<br>Versteegh, Matthijs; Institute for Medical Technology Assessment, Erasmus University Rotterdam,<br>Mavridis, Dimitri; University of Ioannina, Department of Primary Education; Universite Paris Descartes Faculte de Medecine, Sorbone Paris Cité<br>Groene, O; OptiMedis AG, Hamburg, Germany<br>Immonen, Kaisa; European Patients' Forum<br>Wagner, Cordula; Netherlands Institute for Health Services Research, (NIVEL)<br>Canelo-Aybar, Carlos; Iberoamerican Cochrane Centre, CIBERESP-IIB, Sant Pau; Universidad Peruana de Ciencias Aplicadas,<br>Sunol, Rosa; Fundacion Avedis Donabedian, Avedis Donabedian Research Institute (FAD). Universitat Autònoma de Barccelona (UAB); REDISSEC |





| | |
|---|---|
| Keywords: | Protocols & guidelines < HEALTH SERVICES ADMINISTRATION & MANAGEMENT, Public health < INFECTIOUS DISEASES, QUALITATIVE RESEARCH, STATISTICS & RESEARCH METHODS, HEALTH ECONOMICS |
| | |



SCHOLARONE™
Manuscripts

# Comparing the effectiveness and cost-effectiveness of self-management interventions in four high-priority chronic conditions in Europe (COMPAR-EU): a research protocol

**Authors:** Ballester, M; Orrego, C; Heymans, M; Alonso-Coello, P; Versteegh, M; Mavridis, D; Groene, G; Immonen, K; Wagner, C; Canelo, C; and Suñol, S.

**Corresponding author: Ballester, M** (Postal address: Carrer Provença 293, pral.; email: mballester@fadq.org: Telephone number: 0034 934593864)

For peer review only

## ABSTRACT

**Introduction:** Population ageing and increasing chronic illness burden has sparked interest in innovative care models. While self-management interventions (SMIs) are drawing increasing attention, evidence of their efficacy is mostly based on pairwise meta-analysis, generally derived from randomized controlled trials comparing interventions vs. a control or no intervention. As such, relevant efficacy data for comparisons among different SMIs that can be applied to specific chronic conditions is missing. Therefore, the contribution of the currently available evidence to aid decision-making at clinical, organisational, and policy levels is limited.

**Aim:** to identify, compare, and rank the most effective and cost-effective SMIs for adults with four high-priority chronic conditions: type 2 diabetes, obesity, chronic obstructive pulmonary disease, and heart failure.

**Methods and analysis**: All activities will be conducted as part of the COMPAR-EU Project, an EU-funded project designed to bridge the gap between current knowledge and practice on SMIs. In the first phase of the project, we will develop and validate a taxonomy, and a Core Outcome Set (COS) for each condition. These activities will inform a series of systematic review and network meta-analysis (NMA) about the effectiveness of SMIs. We will also perform a cost-effectiveness analysis of the most effective SMIs and an evaluation of contextual factors. We will finally develop tailored decision-making tools for the different relevant stakeholders.

**Ethics and dissemination:** Ethical approval was obtained from the local ethics committee (University Institute for Primary Care Research - IDIAP Jordi Gol). All patients and other stakeholders will provide informed consent prior to participation. This project has been funded by the EU Horizon 2020 research and innovation programme (grant agreement no. 754936). Results will be of interest to relevant stakeholder groups (patients, professionals, managers, policymakers and industry), and will be disseminated in a tailored multi-pronged approach that will include deployment of an interactive platform.

**Keywords:** Protocols & guidelines; Statistics & research methods; Qualitative research; Health economics; Public health.

For peer review only



For peer review only - http://bmjopen.bmj.com/site/about/guidelines.xhtml

## ARTICLE SUMMARY

**Strengths and limitations of this study:**

1. The project will result in the largest NMA of complex SMI interventions.
2. SMIs are inconsistently defined across the literature potentially generating a high level of heterogeneity for the NMA, which we will mitigate by developing a validated a taxonomy.
3. The development of COSs with input from patients and other stakeholders for each chronic condition will ensure that outcomes assessed in the NMA are relevant to the target users.
4. The comparative effectiveness analysis via NMA, cost-effectiveness, and contextual factors evaluation will provide new knowledge that should facilitate future implementation of successful SMIs.
5. An interactive platform will facilitate access to decision making tools relevant to the specific needs of the different target users.

For peer review only



For peer review only - http://bmjopen.bmj.com/site/about/guidelines.xhtml

## INTRODUCTION

As population ageing accelerates worldwide, chronic illness will place an increasing burden on society and healthcare systems (1). Chronic conditions affect over 80% of people aged over 65 in Europe and account for an estimated 77% of disease burden, as measured by disability-adjusted life years (2). Furthermore, between 70% and 80% of healthcare costs in Europe can be attributed to chronic disease, and the current €700 billion expenditure is expected to rise (3).

Self-management support has become a key strategy for addressing chronic disease burden (4), contributing to the paradigm shift from a paternalistic model where patients are viewed as passive recipients of care, towards more equitable and collaborative models of clinician-patient interaction (5).

COMPAR-EU is an EU-funded project designed to bridge the gap between current knowledge and practice on self-management interventions (SMIs). For the purpose of this project self- management is what individuals, families, and communities do to promote, maintain, or restore health and cope with illness and disability, with or without the support of health professionals, and including but not limited to self-prevention, self-diagnosis, self-medication, and coping with illness and disability (6).

Self-management of a chronic condition requires self-efficacy, largely understood as a person's confidence in their ability to cope with their illness (7). To be self-efficacious, people need special skills to cope with the consequences of the disease, including monitoring symptoms and clinical markers, understanding the implications of these, and adjusting behaviours treatment accordingly.

SMIs are supportive interventions systematically delivered or led by healthcare staff or other patients with the aim of building patients' confidence and equipping them with the necessary skills. Their purpose is to actively engage patients (and informal caregivers where appropriate) in the management of their disease (8). As such, they are more than merely didactic, instructional programmes, as their primary objective is to bring about changes in behaviour and trigger a sequence of positive knock-on effects (9).



For peer review only - http://bmjopen.bmj.com/site/about/guidelines.xhtml

SMIs are complex interventions (9), typically characterised by multiple factors (components, formats, settings, target behaviours...) that interact over time as participants move back and forth between the intervention processes and everyday life, which can entail challenges in measuring effectiveness. Despite this, there is promising evidence that SMIs, under given conditions, can improve clinical outcomes in numerous chronic conditions, such as diabetes (reduction of haemoglobin A1c levels) (10), obesity (reduction of weight loss) (11,12), chronic obstructive pulmonary disease (COPD) (improvement of dyspnoea) (13), and heart failure (reduction of mortality) (14). SMIs have also been associated with improvements in patient-reported outcomes, such as quality of life, and more specific disease measures (15), such as self-efficacy (16) and adherence (17).

Evidence on the efficacy of SMIs to date has mostly come from systematic reviews that have employed pairwise meta-analysis of RCTs. Systematic reviews pool evidence from RCTs comparing the same interventions and have long been considered as the highest standard in evidence-based health care. Pairwise meta-analysis, however, leaves a crucial gap, because it requires the RCT's that are pooled to have included the same interventions. To provide decision-makers, clinicians, and patients with solid evidence on how effective an SMI is for a given outcome and disease, multiple interventions need to be compared. In COMPAR-EU, we plan to do this by using NMA to assess the relative effectiveness of SMIs in four chronic conditions.

NMA synthesises direct and indirect evidence across a network of multiple interventions. This method has numerous advantages: it provides more precise effect estimates, allows for the estimation of relative effectiveness between interventions that have not been compared directly, and provides a ranking of interventions by effectiveness, presenting thus a potential analytical advantage (18–22).NMA has been in use for some years, an empirical evaluation of 456 NMAs published up to 2015 showed that just 16% of these addressed complex interventions (23) such as SMIs (24).

While NMA allows us to compare different types of interventions within a health condition, it cannot aid in informing effectiveness across conditions. We know, for example, that certain SMIs, such as telemonitoring has positive effects on lipids levels



For peer review only - http://bmjopen.bmj.com/site/about/guidelines.xhtml

for patients with diabetes (25), and on adherence for patients with heart failure(26) but the NMA can't answer which one of these interventions should be reimbursed from a finite health care budget. Furthermore, a synthesis of trial data is by definition limited to the duration of the trial, while often good estimates of long-term effects (such as long-term weight loss) are needed for societal decision-making.

To address these issues, we will develop simulation models in the four disease areas which will translate benefits as identified by the NMA into long term health benefits expressed in quality adjusted life years (QALYs), which enables us to compare not only SMIs within the four conditions but also across them. The simulation models will also include a cost-effectiveness component. Cost-effectiveness analyses are important as they help to prioritise health expenditure. There is evidence that certain SMIs are cost-effective. Weight reduction in obesity, for example, can produce short-term savings and increase the chances of remaining in employment (27), resulting in additional societal gains, while COPD management interventions can improve quality of life at generally acceptable societal costs (28), and in some cases even result in short-term healthcare cost savings (29). Secondary cardiovascular risk prevention programmes have the potential to reduce direct healthcare expenditure and improve health outcomes (30). While these results have not yet been structured into a consolidated body of knowledge, they do indicate that investing in SMIs may be cost-effective.

A better understanding of facilitators and barriers to successful programme implementation is also essential. Contextual factors at various levels (patient, patient-provider interaction, organisation and system) can all influence SMI uptake, engagement, and success. Improving our understanding of how these factors influence the effectiveness and cost-effectiveness of complex SMIs for chronic illness is much needed.

*Aim*

COMPAR-EU is a multimethod interdisciplinary project that will run from 2018 to 2022. The project has been designed to help bridge the gap between current knowledge and practice in SMIs for chronic illness. Its aim is to identify, compare, and rank the most



For peer review only - http://bmjopen.bmj.com/site/about/guidelines.xhtml

effective SMIs for adults living with four high-priority chronic conditions (type 2 diabetes, obesity, COPD, and heart failure), and among these interventions, categorise the most cost-effective and feasible SMIs. The results of the project will facilitate informed decision-making and support the implementation of best practices in different healthcare contexts through an interactive platform featuring decision-making tools, and other end products adapted to the needs of a range of end users, such as policymakers, guideline developers, researchers, healthcare professionals, patients, and industry.

The specific objectives are to: 1) validate a taxonomy of SMIs; 2) identify and prioritise SMI outcomes from the perspective of both patients and practitioners, culminating in a Core Outcome Set (COS) for each condition; 3) carrying out systematic reviews to synthesise existing evidence on SMIs from RCTs; 4) compare the relative effectiveness of SMIs through NMA; 5) model the cost-effectiveness impact of SMIs; 6) analyse contextual and implementation factors, and; 7) develop and pilot decision-making tools to facilitate access to and use of the most effective SMIs among key target end users.

## METHODS AND ANALYSIS

The COMPAR-EU project is divided into seven phases following to our specific objectives. Each phase is further described below and in Figure 1 (COMPAR-EU Phases and main tasks)

*(Figure 1. COMPAR-EU Phases and main tasks)*

### Phase 1: Refinement and validation of a taxonomy of SMIs

Taxonomies are formal systems for classifying multifaceted, complex phenomena according to a set of common conceptual domains and dimensions; their use increases clarity in defining and comparing complex phenomena (31). Several taxonomies for SMIs have been employed in the literature, but so far have focused in a specific area of the intervention (e.g. self-management behaviour) and haven't been validated. In the first phase of COMPAR-EU, we will produce a conceptual map and classification system that will be evaluated by self-management experts and stakeholders (including patients)



For peer review only - http://bmjopen.bmj.com/site/about/guidelines.xhtml

through a modified Delphi technique consisting of a two-rounds online survey. The resulting feedback and suggestions for refinement will be integrated into a new version of a self-management taxonomy to be tested by the research team when classifying the SMIs reported in the analysed RCTs.

**Phase 2: Develop Core Outcome Sets for SMIs addressing the four conditions**

Interventions can only be compared across studies when they share at least some common outcomes, and appropriate selection of outcomes is essential if research is to guide decision-making and inform policy. We will therefore develop a COS for each of the four chronic conditions included in the project.

- *Outcome catalogue*

The first step is to create an exhaustive database listing outcomes reported in previous EU projects (PRO-STEP (32), and EMPATHIE (33)) and COMET, a COS database and other relevant organisations (using a snowballing technique). For each condition, outcomes will be classified into different categories, such as clinical outcomes, patient-reported outcome measures, or resource utilisation.

- *Systematic review of how patients value self-management outcomes*

To identify patient priorities for self-management in the selected conditions we will conduct a scoping review and a series of specific overviews for each condition about what patients, and their caregivers value on SMIs.

- *Delphi survey with patient representatives*

To help prioritise the outcomes identified, we will use a modified Delphi survey administered to a convenience sample of patients, carers, and patient representatives to ensure that our research addresses outcomes that matter to patients (and other stakeholders). Although patient participation in outcome selection has been studied very little, it has been described as a good practice to minimise the influence of power differentials between stakeholders (34). Four panels of patients and carers, for each disease separately (5-8 members) will be given the task of prioritising relevant outcomes on a Likert scale.

- *Final consensus workshop*

For peer review only



For peer review only - http://bmjopen.bmj.com/site/about/guidelines.xhtml

The proposals for each of the four COSs will be presented to a panel of patients and other stakeholders (healthcare professionals, policymakers and researchers) in a workshop that aims to achieve consensus across groups on the most important outcomes to include in the COS. Participants will be previously provided with the results of the patient prioritization process, and with the results of the synthesis of results from the literature review. We will establish criteria to address potential discrepancies across stakeholders.

**Phase 3: Systematic review – Descriptive synthesis of the evidence**

- *Preparations for data collection*

We will develop a protocol following Cochrane guidance (35,36) and hold training sessions for those responsible for collecting data. Before the extraction process, all reviewers will be trained and undergo calibration to ensure inter-rater agreement. Additional, in the extraction process all data collected will be reviewed by an independent researcher to ensure quality.

- *Literature search and screening*

To identify relevant RCTs, we will draw on the databases of previous European projects (PRO-STEP, and EMPATHIE) that have identified hundreds of systematic reviews on SMIs for diabetes, obesity, COPD, and heart failure. We will then update existing data through new searches in MEDLINE, CINAHL, Embase, Cochrane, and PsycINFO. We will include RCTs that compare SMIs in adults with at least one of our conditions of interest (type 2 diabetes, obesity, COPD, or heart failure) and are published in English or Spanish.

- *Data extraction and collection*

An estimated 4,000 RCTs, based on the results of a previous overview (32), will be studied to extract relevant data on SMI in the four prioritized chronic conditions. These data include among others, patient characteristics, disease characteristics and comorbidities, intervention characteristics (guided by the taxonomy), outcomes (guided by the four COSs), results, and information on study design and risk of bias. Specific attention will be paid to subgroups of patients according to comorbidity, gender, and socioeconomic variables (e.g. health literacy).

- *Descriptive analysis and summary of SMIs and outcomes*



For peer review only - http://bmjopen.bmj.com/site/about/guidelines.xhtml

SMIs and outcomes identified in the RCTs will be described and summarised to provide information on type and number of interventions, outcome results, patient characteristics, and presence of comorbidities for each of the four conditions.

**Phase 4: Systematic review: NMA and certainty of evidence**

- *Network meta-analysis*

We will initially develop theoretical models that make explicit the mechanisms through which the different SMIs operate on a given outcome. Based on our taxonomy of interventions, these models will identify hierarchies of elements (type of support provided, expected self-management behaviour, mode of delivery of the intervention, provider, etc.) that operate on the outcome to identify which components or which combinations of components are most effective.

Additionally to the standard NMA models, we will employ component NMA (37–39) to identify key intervention components and create a ranking of SMIs according to their effectiveness. During this process, it is crucial that major assumptions of NMA like transitivity (that there must be no relevant discrepancy or inconsistency between direct and indirect evidence(40)) are satisfied, as the validity of results will depend on the plausibility of the assumptions made. We will also explore the distribution of effect modifiers (e.g., comorbidities, gender, and socioeconomic factors) across the various comparisons. This will include differentiation, if possible, between the various forms of 'usual care' reported in the included studies.

- *Evaluation and summary of the certainty of the evidence*

To guide users in knowing how much confidence they can place in the summarised evidence; we will rate the certainty of evidence, obtained through the network meta-analysis, for each outcome of interest, using the Grading of Recommendations, Assessment, Development and Evaluations (GRADE) approach(41–43). Additionally, we will apply an alternative approach, the CINeMA framework, to assess the confidence in the results by exploring how information flows in the network and how much studies at high/unclear risk of bias affect the network meta-analysis estimates. We will explore how the assessment differs between these two approaches.

**Phase 5: Model the Cost-effectiveness of effective SMIs**



For peer review only - http://bmjopen.bmj.com/site/about/guidelines.xhtml

For the cost-effectiveness analysis, data on short term effects on intermediate outcomes (e.g. BMI, HbA1C) from the NMA will serve as input for simulation models that extrapolate these effects into long term health effects expressed in Quality Adjusted Life Years (QALYs). These health benefits will be combined with cost prediction to estimate the incremental cost per quality-adjusted life year gained. To develop simulation models in the four chronic conditions the first step will be to develop a conceptual model informed by a review of the literature (44). Decisions will also be taken at this stage on which statistical techniques and models to use (e.g., discrete-event models, Markov models, patient level simulation models). These decisions will be guided by good practice guidelines from pharmacoeconomic communities of medical decision making and the International Society for Pharmacoeconomics and Outcomes Research (45) and available data. In all four disease models, the natural course of each of the four study conditions will be modelled with incorporation of background events and disease-specific mortality and morbidity. Intervention costs will take into account societal, healthcare, and patient perspectives, and where possible, special attention will be given to societal costs that have historically been understudied (e.g., productivity gains and changes in caregiver burden and costs in life-years gained (39). By expressing health benefits in QALYs we will be able to produce a ranking of the most cost-effective SMIs under, within and across conditions.

**Phase 6: Analyse the contextual factors that promote implementation in real life contexts**

For each of the most effective SMIs identified for the four chronic conditions we will perform a realist systematic review (44) to identify key determinants of success (or failure), such as intervention settings (e.g., whether on a primary care level or hospital level care) and mechanisms (e.g., engagement processes) that produce specific outcomes. At the patient level, special attention will be paid to comorbidities, gender, and socioeconomic dimensions such as health literacy to better understand how these influence the implementation of the selected SMIs. We will then use a modified Delphi method to establish the importance of these contextual factors in the target countries (Belgium, Germany, Greece, the Netherlands & Spain,).



For peer review only - http://bmjopen.bmj.com/site/about/guidelines.xhtml

**Phase 7: Development and piloting of the COMPAR-EU information technology platform, and preparation for future implementation**

We will develop a technological platform that will integrate all the information and evidence synthesised during the different phases of the project to facilitate decision making for target end users (patients, healthcare professionals, policymakers, researchers, and small and medium-sized enterprises (SMEs)). The platform will include the following GRADE-based tools:

- Evidence profiles and Interactive Summary of Findings (SoF) tables(45): these presentation will provide information in different formats about the quality of evidence, and magnitude of relative and absolute effects for each of the core outcomes identified.
- Evidence to Decision frameworks (EtD): using semi-automatic templates, interactive EtD frameworks (46) will be completed for a number of priority questions that will take into account the magnitude of desirable and undesirable effects, stakeholder views on the importance of different outcomes, information on resource use and cost-effectiveness, impact on equity, and other aspects like acceptability or feasibility of the interventions. The frameworks will include draft recommendations that could be then applied or adapted to different settings.
- Patient decision aids: will be developed in plain-language for all selected situations identified in the previous phases of the study. The aids will be produced in six languages (English, French, German, Spanish, Dutch, and Greek) and will include information obtained in some of the phases included in the project.

- *Development of COMPAR-EU online platform*

The COMPAR-EU online platform will feature structured interfaces tailored to the needs of different end users. It will offer access to the evidence (including taxonomy of interventions and COSs) and decision-making tools generated during the project and will be designed such that after answering a few simple questions, users will be guided to the tool or product that best suits their needs (Figure 2 illustrates the main products to be integrated into the platform and expected end-users).



For peer review only - http://bmjopen.bmj.com/site/about/guidelines.xhtml

*Figure 2. COMPAR-EU Platform, decision-making tools and other end products*

- *Piloting and refinement of decision-making tools with different stakeholders*

The COMPAR-EU platform will be piloted with end users and relevant stakeholders to gauge potential barriers and understand how to position the decision-making tools in the target healthcare markets. A series of small-scale tests will be used to pilot the decision aids among patients, health professionals, and EtDs and other products with other stakeholders who may be involved in implementing the tools in the five participating countries (e.g., policymakers, industry, and representatives of patient associations). We will organise focus groups and user experience tests in simulated settings using techniques such as thinking-out-loud to gain input for further optimisation of the tools and feedback on the opportunities for implementation in the participating countries and settings.

A group of at least 60 end-users (policymakers, guidelines developers, and researchers depending on the type of tools) will pilot-test? the COMPAR-EU electronic decision-making tools. End-users will be invited to test the tools and give feedback via semi-structured online interviews. The tools will be piloted in different scenarios and countries to identify facilitators and barriers to implementation. Results of the piloting will be used to further improve the tools.

- *Preparation of future implementation*

Even with well-developed and user-friendly decision-tools, integration in existing policy and regulatory frameworks is crucial for successful uptake of our recommendations. We will conduct a systematic search of the scientific and grey literature to identify and analyse the multiple policy and regulatory frameworks that could influence the uptake of the self-management and decision-making tools designed within COMPAR-EU. We will also hold workshops with industry, pharma, and SMEs to identify business opportunities resulting from the research findings that could result in long-term sustainable dissemination.



For peer review only - http://bmjopen.bmj.com/site/about/guidelines.xhtml

## PATIENT AND PUBLIC INVOLVEMENT

Patients are a key component of the COMPAR-EU project from start to finish and their interests are represented by the European Patient Forum (EPF), which is one of the consortium partners. Together with other stakeholders, they will be involved in different aspects of the project including prioritisation of core outcomes and testing of interactive tools. A core group of partners, led by the EPF, will also be created to ensure co-production and establishment of explicit criteria to incorporate patient views into project outcomes, products, and communication interventions. Plain-language material will also be developed to support the main end products of the project.

## ETHICS AND DISSEMINATION

The project coordinator's (Avedis Donabedian Research Institute) requested the overall ethical approval for the project to our local Clinical Research Ethics Committee (CEIC) (the University Institute for Primary Care Research - IDIAP Jordi Gol). Ethical approval was granted on March 2018. Results are of interest to several stakeholder groups (patients, professionals, managers, policymakers and industry) and will be disseminated in a tailored multi-pronged approach, including the creation of an interactive platform. The data generated by the project will be managed following the Golden Open access as defined by the European Commission for Horizon 2020 research projects (47).

For peer review only



For peer review only - http://bmjopen.bmj.com/site/about/guidelines.xhtml

**LIST OF ABBREVIATIONS**

COS – Core Outcome Sets

EPF – European Patient Forum

NMA – Network meta-analysis

SMIs – Self-management interventions

RCT – Randomised Control Trial

For peer review only



For peer review only - http://bmjopen.bmj.com/site/about/guidelines.xhtml

# DECLARATIONS

## PATIENT AND PUBLIC INVOLVEMENT

European Patients Forum (EPF) a key umbrella organization in patient representation and European level is a partner of this project and as such has contributed to the project from its inception and design.

Furthermore we have planned for patient participation in key stages of the project, including the selection of outcomes for the Core Outcome Sets, advise towards patient end products and participating in the design and piloting of the COMPAR-EU platform and related decision making tools.

## AUTHORS' CONTRIBUTIONS

- Marta Ballester contributed to the design of the project and prepared the manuscript of the paper.
- Carola Orrego contributed to the design of the project and reviewed and contributed to the manuscript of the paper.
- Monique Heymans contributed to the design of the project and reviewed and contributed to the manuscript of the paper.
- Pablo Alonso-Coello contributed to the design of the project and reviewed and contributed to the manuscript of the paper.
- Matthijs Versteegh contributed to the design of the project and reviewed and contributed to the manuscript of the paper.
- Dimitris Mavridis contributed to the design of the project and reviewed and contributed to the manuscript of the paper.
- Oliver Groene contributed to the design of the project and reviewed and contributed to the manuscript of the paper.



For peer review only - http://bmjopen.bmj.com/site/about/guidelines.xhtml

- Kaisa Immonen contributed to the design of the project and reviewed and contributed to the manuscript of the paper.
- Cordula Wagner contributed to the design of the project and reviewed and contributed to the manuscript of the paper.
- Carlos Canelo contributed to the design of the project and reviewed and contributed to the manuscript of the paper.
- Rosa Suñol contributed to the design of the project and reviewed and contributed to the manuscript of the paper.

- Acknowledgments As well, we would like to express our gratitude to Ana Isabel González, Aretj-Angeliki Veroniki, Claudia Valli, Claudio Alfonso Rocha Calderón, Ena Niño de Guzmán, Estela Camus, Giorgos Seitidis, Hector Pardo-Hernandez , Jany Rademakers, Jessica Beltrán, Karla Salas, Kevin Pacheco-Barrios, Lyudmil Ninov, Maria Petropoulou, Marieke van der Gaag, Montserrat León, Nina Adrion, Rune Poortvliet, Stella Zevgiti, Valentina Strammiello for their valuable assistance in conducting this proposal.
- Funding: This work was supported by European Union's Horizon 2020 research and innovation programme under grant agreement No 754936
- Competing interest statement: no competing interests.



For peer review only - http://bmjopen.bmj.com/site/about/guidelines.xhtml

## REFERENCES

1. Chang AY, Skirbekk VF, Tyrovolas S, Kassebaum NJ, Dieleman JL. Measuring population ageing: an analysis of the Global Burden of Disease Study 2017. Lancet Public Heal [Internet]. 2019 Mar 1 [cited 2019 Aug 1];4(3):e159–67. Available from: http://www.ncbi.nlm.nih.gov/pubmed/30851869

2. Patient empowerment--who empowers whom? Lancet (London, England) [Internet]. 2012 May 5 [cited 2016 Oct 4];379(9827):1677. Available from: http://www.ncbi.nlm.nih.gov/pubmed/22559881

3. Anderson G, Hopkins J. Responding to the Growing Cost and Prevalence of People With Multiple Chronic Conditions. 2007.

4. Bycroft JJ, Tracey J. Self-management support: Awin-win solution for the 21st century. N Z Fam Physician [Internet]. 2006 [cited 2017 Apr 6];33(4):243–8. Available from: http://koawatea.co.nz/wp-content/uploads/2014/10/Self-Management-support-A-win-win-solution-Bycroft-J-Tracey-J-2006.pdf

5. Aujoulat I, Marcolongo R, Bonadiman L, Deccache A. Reconsidering patient empowerment in chronic illness: A critique of models of self-efficacy and bodily control. Soc Sci Med [Internet]. 2008 Mar [cited 2017 Apr 6];66(5):1228–39. Available from: http://www.ncbi.nlm.nih.gov/pubmed/18155338

6. European Commission. Pilot project on the promotion of self-care in chronic diseases in the European Union [Internet]. [cited 2017 Apr 7]. Available from: http://ec.europa.eu/dgs/health_food-safety/funding/docs/call_sante_2015-d2-021-tech_specs_en.pdf

7. Bandura A. Toward a unifying theory of behavioral change. Psychol Rev [Internet]. 1977;84(2):191–215. Available from: http://www.apa.org/pubs/journals/rev/

8. Department of Health. The Expert Patient: A new approach to chronic disease management for the 21th century. London; 2011.

9. Pigott T, Shepperd S. Identifying, documenting and examining heterogeneity in



For peer review only - http://bmjopen.bmj.com/site/about/guidelines.xhtml

systematic reviews of complex interventions. J Clin Epidemiol. 2013;66:1244 – 1250.

10. Umpierre D, Kramer CK, Leita CB, Gross JL, Ribeiro JP, Schaan BD. Physical Activity Advice Only or Structured exercise training and association With HbA 1c Levels in Type 2 Diabetes. JAMA J Am Med Assoc. 2011;305:1790–9.

11. Armstrong MJ, Mottershead TA, Ronksley PE, Sigal RJ, Campbell TS, Hemmelgarn BR. Motivational interviewing to improve weight loss in overweight and/or obese patients: A systematic review and meta-analysis of randomized controlled trials. Obes Rev. 2011;12(9):709–23.

12. Silveira JAC da, Taddei JA de AC, Guerra PH, Nobre MRC. The effect of participation in school-based nutrition education interventions on body mass index: A meta-analysis of randomized controlled community trials. Prev Med (Baltim). 2013;56(3–4):237–43.

13. McLean S, Nurmatov U. Telehealthcare for chronic obstructive pulmonary disease. Cochrane Libr. 2011;(8).

14. Inglis Sally C, Clark Robyn A, Dierckx R, Prieto-Merino D, Cleland John GF. Structured telephone support or non-invasive telemonitoring for patients with heart failure. Cochrane Database Syst Rev. 2015;(10).

15. Steinsbekk A, Rygg LØ, Lisulo M, Rise MB, Fretheim A. Group based diabetes self-management education compared to routine treatment for people with type 2 diabetes mellitus. A systematic review with meta-analysis. BMC Health Serv Res. 2012 Jan;12(1):213.

16. de Jongh T, Gurol-Urganci I, Vodopivec-Jamsek V, Car J, Atun R. Mobile phone messaging for facilitating self-management of long-term illnesses. Cochrane database Syst Rev. 2012;12(12):CD007459.

17. Tricco AC, Ivers NM, Grimshaw JM, Moher D, Turner L, Galipeau J, et al. Effectiveness of quality improvement strategies on the management of diabetes: A systematic review and meta-analysis. Lancet. 2012;379(9833):2252–61.




For peer review only - http://bmjopen.bmj.com/site/about/guidelines.xhtml

18. Caldwell DM, Ades a E, Higgins JPT. Simultaneous comparison of multiple treatments: combining direct and indirect evidence. BMJ Br Med J. 2005;331(7521):897–900.

19. Salanti G. Indirect and mixed-treatment comparison, network, or multiple-treatments meta-analysis: many names, many benefits, many concerns for the next generation evidence synthesis tool. Res Synth Methods [Internet]. 2012;3(March):80–97. Available from: http://doi.wiley.com/10.1002/jrsm.1037

20. Mavridis D, Giannatsi M, Cipriani A, Salanti G. A primer on network meta-analysis with emphasis on mental health. Evid Based Ment Health [Internet]. 2015 May [cited 2017 Feb 22];18(2):40–6. Available from: http://www.ncbi.nlm.nih.gov/pubmed/25908686

21. Efthimiou O, Debray TPA, van Valkenhoef G, Trelle S, Panayidou K, Moons KGM, et al. GetReal in network meta-analysis: a review of the methodology. Res Synth Methods. 2016;7(3):236–63.

22. Dias S, Welton NJ, Sutton AJ, Ades AE. Evidence Synthesis for Decision Making 3: Heterogeneity—Subgroups, MetaRegression, Bias, and Bias-Adjustment. Med Decis Mak [Internet]. 2013;33(5):597–606. Available from: http://www.pubmedcentral.nih.gov/articlerender.fcgi?artid=3704205&tool=pmcentrez&rendertype=abstract

23. Petropoulou M, Nikolakopoulou A, Veroniki A-A, Rios P, Vafaei A, Zarin W, et al. Bibliographic study showed improving statistical methodology of network meta-analyses published between 1999 and 2015. J Clin Epidemiol [Internet]. 2016; Available from: http://dx.doi.org/10.1016/j.jclinepi.2016.11.002

24. Petticrew M, Rehfuess E, Noyes J et al. Synthesizing evidence on complex interventions: how meta-analytical, qualitative, and mixed-method approaches can contribute. J Clin Epidemiol. 2013;66:1230–43.

25. Ramadas A, Quek KF, Chan CKY, Oldenburg B. Web-based interventions for the management of type 2 diabetes mellitus: A systematic review of recent evidence. Int J Med Inform [Internet]. 2011;80(6):389–405. Available from:

For peer review only



For peer review only - http://bmjopen.bmj.com/site/about/guidelines.xhtml

http://dx.doi.org/10.1016/j.ijmedinf.2011.02.002

26. Radhakrishnan K, Jacelon C. Impact of Telehealth on Patient Self-management of Heart Failure. A Review of Literature. J Cardiovasc Nurs [Internet]. 2012;27(1):33–43. Available from: http://www.ncbi.nlm.nih.gov/pubmed/21558862

27. Reichert AR. Obesity, Weight Loss, and Employment Prospects: Evidence from a Randomized Trial. J Hum Resour. 2015;50(3):759–810.

28. Hoogendoorn M, van Wetering CR, Schols AM, Rutten-van Mölken MPMH. Is INTERdisciplinary COMmunity-based COPD management (INTERCOM) cost-effective? Eur Respir J. 2009;35(1).

29. Boland MR, Tsiachristas A, Kruis AL, Chavannes NH, Rutten-van Mölken MP. The health economic impact of disease management programs for COPD: a systematic literature review and meta-analysis. BMC Pulm Med. 2013 Dec;13(1):40.

30. Tsiachristas A, Burgers L, Rutten-van Mölken MPMH. Cost-Effectiveness of Disease Management Programs for Cardiovascular Risk and COPD in The Netherlands. Value Heal. 2015;18(8):977–86.

31. Bradley EH, Curry LA, Devers KJ. Qualitative data analysis for health services research: Developing taxonomy, themes, and theory. Health Serv Res. 2007;42(4):1758–72.

32. PRO-STEP Consortium. PRO STEP Project [Internet]. Available from: http://ec.europa.eu/dgs/health_food-safety/funding/call_sante_2015-d2-021_en.htm

33. EMPATHiE Consortium. Final summary report: EMPATHiE, Empowering patients in the management of chronic diseases [Internet]. 2014. Available from: http://ec.europa.eu/health//sites/health/files/patient_safety/docs/empathie_frep_en.pdf

34. Kirwan JR, Minnock P, Adebajo A, Bresnihan B, Choy E, De Wit M, et al. Patient perspective: Fatigue as a recommended patient centered outcome measure in rheumatoid arthritis. In: Journal of Rheumatology. 2007. p. 1174–7.

For peer review only



For peer review only - http://bmjopen.bmj.com/site/about/guidelines.xhtml

35. Higgins JP, Green S. Cochrane Handbook for Systematic Reviews of [Internet]. Vol. Version 5., The Cochrane Collaboration. 2008. Version 5.1.0. Available from: http://onlinelibrary.wiley.com/doi/10.1002/9780470712184.fmatter/summary

36. Chaimani A, Caldwell DM, Li T, Higgins JPT, Salanti G. Additional considerations are required when preparing a protocol for a systematic review with multiple interventions. J Clin Epidemiol [Internet]. 2017;83:65–74. Available from: http://dx.doi.org/10.1016/j.jclinepi.2016.11.015

37. Caldwell DM, Welton NJ. Approaches for synthesising complex mental health interventions in meta-analysis. Evid Based Ment Health [Internet]. 2016 Feb 1 [cited 2019 Mar 8];19(1):16–21. Available from: http://www.ncbi.nlm.nih.gov/pubmed/26792834

38. Freeman SC, Scott NW, Powell R, Johnston M, Sutton AJ, Cooper NJ. Component network meta-analysis identifies the most effective components of psychological preparation for adults undergoing surgery under general anesthesia. J Clin Epidemiol [Internet]. 2018 Jun 1 [cited 2019 Mar 8];98:105–16. Available from: https://www.sciencedirect.com/science/article/pii/S089543561731137X

39. Welton NJ, Caldwell DM, Adamopoulos E, Vedhara K. Mixed Treatment Comparison Meta-Analysis of Complex Interventions: Psychological Interventions in Coronary Heart Disease. Am J Epidemiol [Internet]. 2009 May 1 [cited 2019 Mar 8];169(9):1158–65. Available from: http://www.ncbi.nlm.nih.gov/pubmed/19258485

40. Tonin FS, Rotta I, Mendes AM, Pontarolo R, Tonin FS, Rotta I, et al. Network meta-analysis: a technique to gather evidence from direct and indirect comparisons. Pharm Pract (Granada) [Internet]. 2017 Mar 31 [cited 2019 Aug 2];15(1):943–943. Available from: http://www.pharmacypractice.org/journal/index.php/pp/article/view/943/490

41. Yepes-Nuñez JJ, Li S-A, Guyatt G, Jack SM, Brozek JL, Beyene J, et al. Development of the summary of findings table for network meta-analysis. J Clin Epidemiol [Internet]. 2019 Nov 2 [cited 2019 Oct 1];115:1–13. Available from:

For peer review only



For peer review only - http://bmjopen.bmj.com/site/about/guidelines.xhtml

http://www.ncbi.nlm.nih.gov/pubmed/31055177

42. Brignardello-Petersen R, Bonner A, Alexander PE, Siemieniuk RA, Furukawa TA, Rochwerg B, et al. Advances in the GRADE approach to rate the certainty in estimates from a network meta-analysis. J Clin Epidemiol [Internet]. 2018 Jan [cited 2019 Oct 1];93:36–44. Available from: http://www.ncbi.nlm.nih.gov/pubmed/29051107

43. Guyatt GH, Oxman AD, Schünemann HJ, Tugwell P, Knottnerus A. GRADE guidelines: A new series of articles in the Journal of Clinical Epidemiology. J Clin Epidemiol [Internet]. 2011 Apr [cited 2019 Oct 1];64(4):380–2. Available from: http://www.ncbi.nlm.nih.gov/pubmed/21185693

44. Pawson R, Greenhalgh T, Harvey G, Al E. Realist review- a new method of systematic review designed for complex policies interventions. J Heal Serv Res Policy. 2005;(10):21–34.

45. Guyatt G, Oxman AD, Akl EA, Kunz R, Vist G, Brozek J, et al. GRADE guidelines: 1. Introduction—GRADE evidence profiles and summary of findings tables. J Clin Epidemiol [Internet]. 2011 Apr [cited 2019 Oct 1];64(4):383–94. Available from: http://www.ncbi.nlm.nih.gov/pubmed/21195583

46. Alonso-Coello P, Schünemann HJ, Moberg J, Brignardello-Petersen R, Akl EA, Davoli M, et al. GRADE Evidence to Decision (EtD) frameworks: a systematic and transparent approach to making well informed healthcare choices. 1: Introduction. BMJ [Internet]. 2016 Jun 28 [cited 2019 Oct 1];353:i2016. Available from: https://www.bmj.com/content/353/bmj.i2016

47. Guidelines to the Rules on Open Access to Scientific Publications and Open Access to Research Data in Horizon 2020 [Internet]. 2017. Available from: https://ec.europa.eu/research/participants/data/ref/h2020/grants_manual/hi/oa_pilot/h2020-hi-oa-pilot-guide_en.pdf

For peer review only



For peer review only - http://bmjopen.bmj.com/site/about/guidelines.xhtml

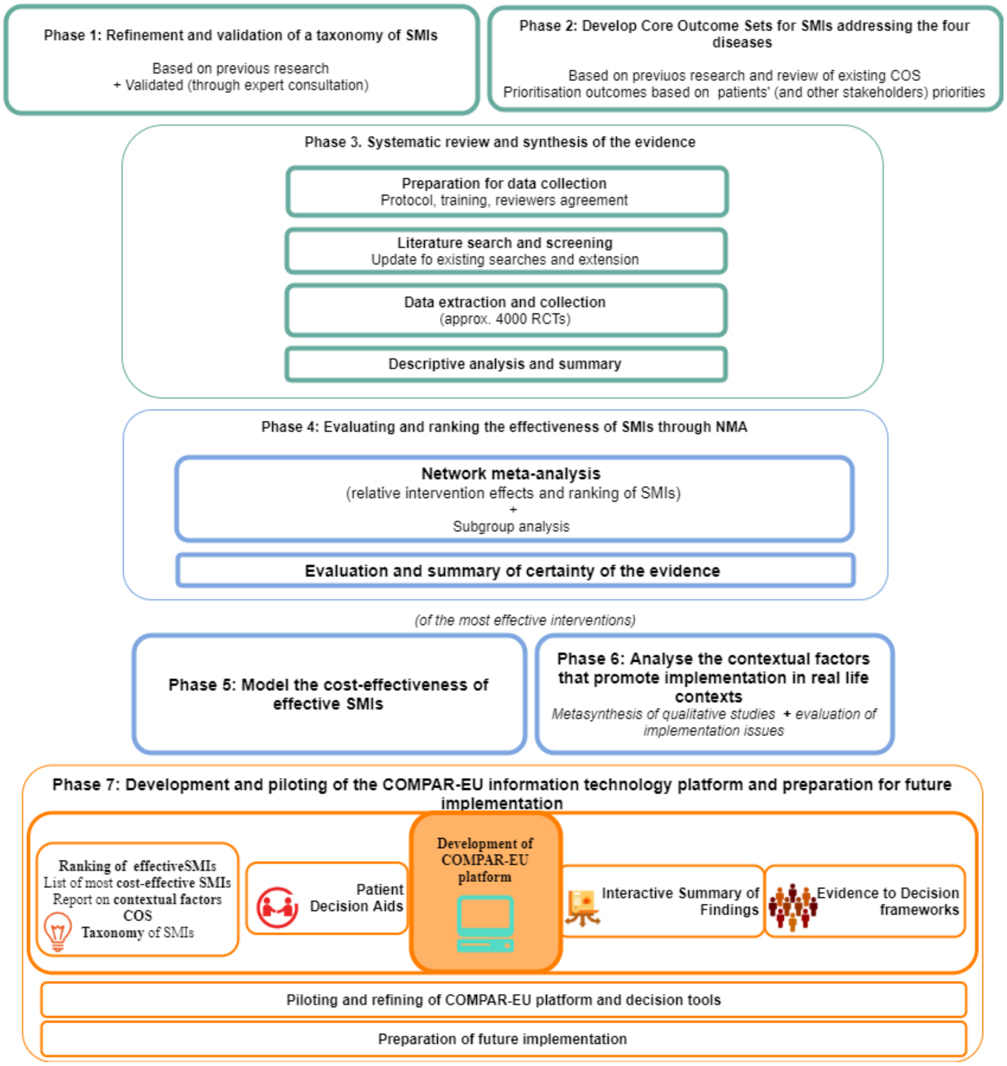


COMPAR-EU Main tasks

297x314mm (72 x 72 DPI)

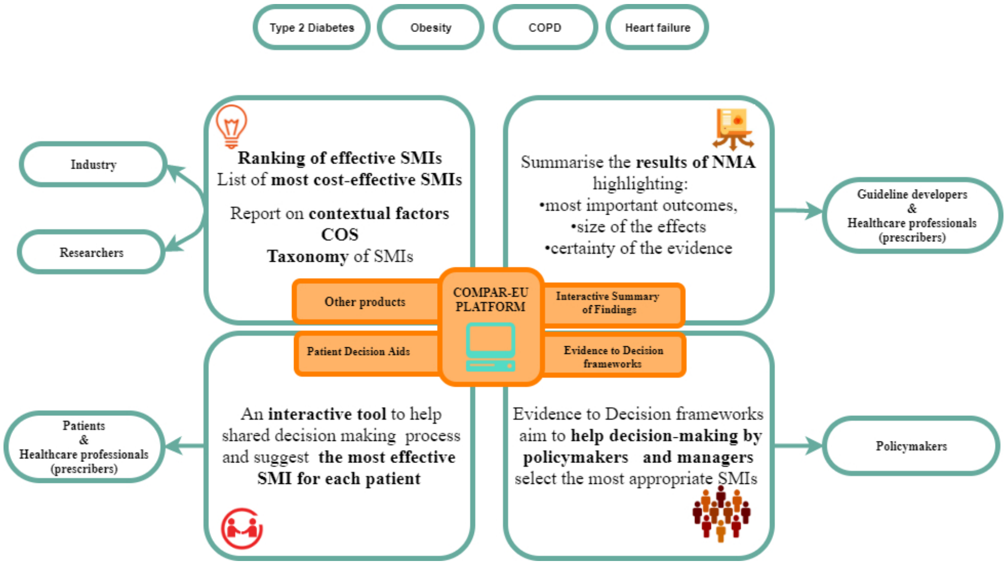


COMPAR-EU online platform and relation to target end-users

343x191mm (72 x 72 DPI)

BMJ Open

# Comparing the effectiveness and cost-effectiveness of self-management interventions in four high-priority chronic conditions in Europe (COMPAR-EU): a research protocol

| | |
|---|---|
| Journal: | *BMJ Open* |
| Manuscript ID | bmjopen-2019-034680.R1 |
| Article Type: | Protocol |
| Date Submitted by the Author: | 10-Dec-2019 |
| Complete List of Authors: | Ballester, Marta; Fundacion Avedis Donabedian, Avedis Donabedian Research Institute (FAD). Universitat Autònoma de Barcelona (UAB); REDISSEC<br>Orrego, Carola; Fundacion Avedis Donabedian, Avedis Donabedian Research Institute (FAD). Universitat Autònoma de Barcelona (UAB); REDISSEC<br>Heijmans, Monique; Netherlands Institute for Health Services Research, (NIVEL)<br>Alonso-Coello, Pablo; Iberoamerican Cochrane Centre, CIBERESP-IIB Sant Pau,<br>Versteegh, Matthijs; Institute for Medical Technology Assessment, Erasmus University Rotterdam,<br>Mavridis, Dimitri; University of Ioannina, Department of Primary Education; Universite Paris Descartes Faculte de Medecine, Sorbone Paris Cité<br>Groene, O; OptiMedis AG, Hamburg, Germany<br>Immonen, Kaisa; European Patients' Forum<br>Wagner, Cordula; Netherlands Institute for Health Services Research, (NIVEL)<br>Canelo-Aybar, Carlos; Iberoamerican Cochrane Centre, CIBERESP-IIB, Sant Pau; Universidad Peruana de Ciencias Aplicadas,<br>Sunol, Rosa; Fundacion Avedis Donabedian, Avedis Donabedian Research Institute (FAD). Universitat Autònoma de Barcelona (UAB); REDISSEC |
| <b>Primary Subject Heading</b>: | Research methods |
| Secondary Subject Heading: | Evidence based practice, Global health, Health economics, Health policy |
| Keywords: | Protocols & guidelines < HEALTH SERVICES ADMINISTRATION & MANAGEMENT, Public health < INFECTIOUS DISEASES, QUALITATIVE RESEARCH, STATISTICS & RESEARCH METHODS, HEALTH ECONOMICS |
| | |

SCHOLARONE™
Manuscripts

# Comparing the effectiveness and cost-effectiveness of self-management interventions in four high-priority chronic conditions in Europe (COMPAR-EU): a research protocol

**Authors:** Ballester, M; Orrego, C; Heijmans, M; Alonso-Coello, P; Versteegh, M; Mavridis, D; Groene, O; Immonen, K; Wagner, C; Canelo, C; and Sunol, R for the COMPAR-EU consortium

**Corresponding author: Ballester, M** (Postal address: Carrer Provença 293, pral.; email: mballester@fadq.org: Telephone number: 0034 934593864)



For peer review only - http://bmjopen.bmj.com/site/about/guidelines.xhtml

## ABSTRACT

**Introduction:** Population ageing and increasing chronic illness burden has sparked interest in innovative care models. While self-management interventions (SMIs) are drawing increasing attention, evidence of their efficacy is mostly based on pairwise meta-analysis, generally derived from randomized controlled trials comparing interventions vs. a control or no intervention. As such, relevant efficacy data for comparisons among different SMIs that can be applied to specific chronic conditions is missing. Therefore, the relevance of the available evidence for decision-making at clinical, organisational, and policy levels is limited.

**Aim:** to identify, compare, and rank the most effective and cost-effective SMIs for adults with four high-priority chronic conditions: type 2 diabetes, obesity, chronic obstructive pulmonary disease, and heart failure.

**Methods and analysis**: All activities will be conducted as part of the COMPAR-EU Project, an EU-funded project designed to bridge the gap between current knowledge and practice on SMIs. In the first phase of the project, we will develop and validate a taxonomy, and a Core Outcome Set (COS) for each condition. These activities will inform a series of systematic review and network meta-analysis (NMA) about the effectiveness of SMIs. We will also perform a cost-effectiveness analysis of the most effective SMIs and an evaluation of contextual factors. We will finally develop tailored decision-making tools for the different relevant stakeholders.

**Ethics and dissemination:** Ethical approval was obtained from the local ethics committee (University Institute for Primary Care Research - IDIAP Jordi Gol). All patients and other stakeholders will provide informed consent prior to participation. This project has been funded by the EU Horizon 2020 research and innovation programme (grant agreement no. 754936). Results will be of interest to relevant stakeholder groups (patients, professionals, managers, policymakers and industry), and will be disseminated in a tailored multi-pronged approach that will include deployment of an interactive platform.


**Keywords:** Protocols & guidelines; Statistics & research methods; Qualitative research; Health economics; Public health.



For peer review only - http://bmjopen.bmj.com/site/about/guidelines.xhtml

## ARTICLE SUMMARY

**Strengths and limitations of this study:**

1. The project will result in the largest NMA of complex SMI interventions.
2. SMIs are inconsistently defined across the literature potentially generating a high level of heterogeneity for the NMA, which we will mitigate by developing a validated taxonomy.
3. The development of COSs with input from patients and other stakeholders for each chronic condition will ensure that outcomes assessed in the NMA are relevant to the target users.
4. The comparative effectiveness analysis via NMA, cost-effectiveness, and contextual factors evaluation will provide new knowledge that should facilitate future implementation of successful SMIs.
5. An interactive platform will facilitate access to decision making tools relevant to the specific needs of the different target users.

For peer review only



For peer review only - http://bmjopen.bmj.com/site/about/guidelines.xhtml

## INTRODUCTION

As population ageing accelerates worldwide, chronic illness will place an increasing burden on society and healthcare systems (1). Chronic conditions affect over 80% of people aged over 65 in Europe and account for an estimated 77% of disease burden, as measured by disability-adjusted life years (2). Furthermore, between 70% and 80% of healthcare costs in Europe can be attributed to chronic disease, and the current €700 billion expenditure is expected to rise (3).

Self-management support has become a key strategy for addressing chronic disease burden (4), contributing to the paradigm shift from a paternalistic model where patients are viewed as passive recipients of care, towards more equitable and collaborative models of clinician-patient interaction (5).

COMPAR-EU is an EU-funded project designed to bridge the gap between current knowledge and practice on self-management interventions (SMIs). For the purpose of this project we define self- management as "actions that individuals, families, and communities engage in to promote, maintain, or restore health and cope with illness and disability, with or without the support of health professionals, and including but not limited to self-prevention, self-diagnosis, self-medication, and coping with illness and disability" (6).

Self-management of a chronic condition requires self-efficacy, largely understood as a person's confidence in their ability to cope with their illness (7). To be self-efficacious, people need special skills to cope with the consequences of the disease, including monitoring symptoms and clinical markers, understanding the implications of these, and adjusting behaviours treatment accordingly.

SMIs are supportive interventions systematically delivered or led by healthcare staff or other patients with the aim of building patients' confidence and equipping them with the necessary skills. Their purpose is to actively engage patients (and informal caregivers where appropriate) in the management of their disease (8). As such, they are more than merely didactic, instructional programmes, as their primary objective is



For peer review only - http://bmjopen.bmj.com/site/about/guidelines.xhtml

to bring about changes in behaviour and trigger a sequence of positive knock-on effects (9).

SMIs are complex interventions (9), typically characterised by multiple factors (components, formats, settings, target behaviours...) that interact over time as participants move back and forth between the intervention processes and everyday life, which can entail challenges in measuring effectiveness. Despite this, there is promising evidence that SMIs, under given conditions, can improve clinical outcomes in numerous chronic conditions, such as diabetes (reduction of haemoglobin A1c levels) (10), obesity (reduction of weight loss) (11,12), chronic obstructive pulmonary disease (COPD) (improvement of dyspnoea) (13), and heart failure (reduction of mortality) (14). SMIs have also been associated with improvements in patient-reported outcomes, such as quality of life, and more specific disease measures (15), such as self-efficacy (16) and adherence (17).

Evidence on the efficacy of SMIs to date has mostly come from systematic reviews that have employed pairwise meta-analysis of RCTs. Systematic reviews pool evidence from RCTs comparing the same interventions and have long been considered as the highest standard in evidence-based health care. Pairwise meta-analysis, however, leaves a crucial gap, because it requires the RCT's that are pooled to have included the same interventions. To provide decision-makers, clinicians, and patients with solid evidence on how effective an SMI is for a given outcome and disease, multiple interventions need to be compared. In COMPAR-EU, we plan to do this by using NMA to assess the relative effectiveness of SMIs in four chronic conditions.

NMA synthesises direct and indirect evidence across a network of multiple interventions. This method has numerous advantages: it provides more precise effect estimates, allows for the estimation of relative effectiveness between interventions that have not been compared directly, and provides a ranking of interventions by effectiveness, presenting thus a potential analytical advantage (18–22). NMA has been in use for some years, an empirical evaluation of 456 NMAs published up to 2015 showed that just 16% of these addressed complex interventions (23) such as SMIs (24).



For peer review only - http://bmjopen.bmj.com/site/about/guidelines.xhtml

To support future reimbursement decisions based on estimates of the long-term effects of SMIs, we will develop simulation models in the four disease areas which will translate benefits as identified by the NMA into long term health benefits expressed in quality adjusted life years (QALYs), which enables us to compare not only SMIs within but also across the four conditions. The simulation models will also include a cost-effectiveness component. Cost-effectiveness analyses are important as they help to prioritise health expenditure. There is evidence that certain SMIs are cost-effective. Weight reduction in obesity, for example, can produce short-term savings and increase the chances of remaining in employment (25), resulting in additional societal gains, while COPD management interventions can improve quality of life at generally acceptable societal costs (26), and in some cases even result in short-term healthcare cost savings (27). Secondary cardiovascular risk prevention programmes have the potential to reduce direct healthcare expenditure and improve health outcomes (28). While these results have not yet been structured into a consolidated body of knowledge, they do indicate that investing in SMIs may be cost-effective.

A better understanding of facilitators and barriers to successful programme implementation is also essential. Contextual factors at various levels (patient, patient-provider interaction, organisation and system) can all influence SMI uptake, engagement, and success. Improving our understanding of how these factors influence the effectiveness and cost-effectiveness of complex SMIs for chronic illness is much needed.

*Aim*

COMPAR-EU is a multimethod interdisciplinary project that will run from 2018 to 2022. The project has been designed to help bridge the gap between current knowledge and practice in SMIs for chronic illness. Its aim is to identify, compare, and rank the most effective SMIs for adults living with four high-priority chronic conditions (type 2 diabetes, obesity, COPD, and heart failure), and among these interventions, categorise the most cost-effective and feasible SMIs. The results of the project will facilitate informed decision-making and support the implementation of best practices in different healthcare contexts through an interactive platform featuring decision-



For peer review only - http://bmjopen.bmj.com/site/about/guidelines.xhtml

making tools, and other end products for policymakers, guideline developers, researchers, healthcare professionals, patients, and industry.

The specific objectives are to: 1) validate a taxonomy of SMIs; 2) identify and prioritise SMI outcomes from the perspective of both patients and practitioners, culminating in a Core Outcome Set (COS) for each condition; 3) carrying out systematic reviews to synthesise existing evidence on SMIs from RCTs; 4) compare the relative effectiveness of SMIs through NMA; 5) model the cost-effectiveness impact of SMIs; 6) analyse contextual and implementation factors, and; 7) develop and pilot decision-making tools to facilitate access to and use of the most effective SMIs among key target end users.

## METHODS AND ANALYSIS

The COMPAR-EU project is divided into seven phases following to our specific objectives. Each phase is further described below and in Figure 1 (COMPAR-EU Phases and main tasks)

*(Figure 1. COMPAR-EU Phases and main tasks)*

### Phase 1: Refinement and validation of a taxonomy of SMIs

Taxonomies are formal systems for classifying multifaceted, complex phenomena according to a set of common conceptual domains and dimensions; their use increases clarity in defining and comparing complex phenomena (29). Several taxonomies for SMIs have been developed in the literature, but so far have focused in a specific area of the intervention (e.g. self-management behaviour) and haven't been validated. In the first phase of COMPAR-EU, we will produce a conceptual map and classification system that will be evaluated by self-management experts and stakeholders (including patients) through a modified Delphi technique consisting of a two-rounds online survey. The list of candidate participants will include authors on self-management or related topics taxonomies, professional experts in self-management and patient representatives. The resulting feedback and suggestions for refinement will be



For peer review only - http://bmjopen.bmj.com/site/about/guidelines.xhtml

integrated into a new version that will be tested by the research team when classifying the SMIs reported in the RCTs that will be included in the NMA.

**Phase 2: Development of Core Outcome Sets for each condition**

Interventions can only be compared across studies when they share at least some common outcomes, and appropriate selection of outcomes is essential if research is to guide decision-making and inform policy. We will therefore develop a COS for each of the four chronic conditions included in the project.

- *Outcome catalogue*

The first step is to create an exhaustive database listing outcomes reported in previous EU projects (PRO-STEP (30), and EMPATHIE (31)) and COMET, a COS database and other relevant organisations (using a snowballing technique). For each condition, outcomes will be classified into different categories, such as clinical outcomes, patient-reported outcome measures, or resource utilisation.

- *Systematic review of how patients value self-management outcomes*

To identify patient priorities for self-management in the selected conditions we will conduct a scoping review, of quantitative and qualitative studies, and a series of specific overviews for each condition about what patients, and their caregivers' value on SMIs.

- *Delphi survey with patient representatives*

We plan to include patient participation in our outcome selection as it has been described as a good practice to minimise the influence of power differentials between stakeholders (32). We will use a modified Delphi survey administered to a convenience sample of patients, and patient representatives to ensure that our research addresses outcomes that matter to patients (and other stakeholders). Four panels of patients and carers, for each disease separately (5-8 members) will be given the task of prioritising relevant outcomes on a Likert scale.

- *Consensus workshop*

The proposals for each of the four COSs will be presented to a panel of patients and other stakeholders (healthcare professionals, policymakers and researchers) in a workshop that aims to achieve consensus across groups on the most important outcomes to include in each COS.



For peer review only - http://bmjopen.bmj.com/site/about/guidelines.xhtml

Participants will be previously provided with the results of the patient prioritization process, and with the results of the synthesis of results from the literature review. We will establish criteria to address potential discrepancies across stakeholders.

**Phase 3: Systematic review – Descriptive synthesis of the evidence**

- *Preparations for data collection*

We will develop a protocol following Cochrane guidance (33,34) and hold training sessions for those responsible for collecting data. Before the extraction process, all reviewers will be trained and undergo calibration to ensure inter-rater agreement. Additional, in the extraction process all data collected will be reviewed by an independent researcher to ensure quality.

- *Literature search and screening*

To identify relevant RCTs, we will draw on the databases of previous European project (PRO-STEP) that identified hundreds of systematic reviews on SMIs for diabetes, obesity, COPD, and heart failure. We will use these RCTs published from 2000 up to 2015 (last date of SR publication included in those projects) in our project, and update this data set through new searches in MEDLINE, CINAHL, Embase, Cochrane, and PsycINFO. We will include RCTs that compared SMIs in adults with at least one of our conditions of interest (type 2 diabetes, obesity, COPD, or heart failure) and are published in English or Spanish. The search will be focused in RCTs published from 2015 to 2018 to complement the findings of the SR included in PRO-STEP, with the possibility of including previous years if the systematic reviews from PRO-STEP haven't covered those previous years sufficiently. An total estimate of 4,000 RCTs, based on the results of a previous overview (30), will be included for the four prioritized chronic conditions.

- *Data extraction and collection*

We will extract data including: patient characteristics, disease characteristics and comorbidities, intervention characteristics (guided by the taxonomy), outcomes (guided by the four COSs), results, and information on study design and risk of bias. Specific attention will be paid to subgroups of patients according to comorbidity, gender, and socioeconomic variables (e.g. health literacy).

- *Descriptive analysis and summary of SMIs and outcomes*



For peer review only - http://bmjopen.bmj.com/site/about/guidelines.xhtml

SMIs and outcomes identified in the RCTs will be described and summarised to provide information on type and number of interventions, outcome results, patient characteristics, and presence of comorbidities for each of the four conditions.

**Phase 4: Systematic review: NMA and certainty of evidence**

- *Network meta-analysis*

We will initially develop theoretical models that make explicit the mechanisms through which the different SMIs operate on a given outcome. Based on our taxonomy of interventions, these models will identify hierarchies of elements (type of support provided, expected self-management behaviour, mode of delivery of the intervention, provider, etc.) that operate on the outcome to identify which components or which combinations of components are most effective.

Additionally to the standard NMA models, we will employ component NMA (35–37) to identify key intervention components and create a ranking of SMIs according to their effectiveness. During this process, it is crucial that major assumptions of NMA like transitivity (that there must be no relevant discrepancy or inconsistency between direct and indirect evidence(38)) are satisfied, as the validity of results will depend on the plausibility of the assumptions made. We will also explore the distribution of effect modifiers (e.g., comorbidities, gender, and socioeconomic factors) across the various comparisons. This will include differentiation, if possible, between the various forms of 'usual care' reported in the included studies.

- *Evaluation and summary of the certainty of the evidence*

To guide users in knowing how much confidence they can place in the summarised evidence; we will rate the certainty of evidence, obtained through the network meta-analysis, for each outcome of interest, using the Grading of Recommendations, Assessment, Development and Evaluations (GRADE) approach(39–41). Additionally, we will apply an alternative approach, the CINeMA framework, to assess the confidence in the results by exploring how information flows in the network and how much studies at high/unclear risk of bias affect the network meta-analysis estimates. We will explore how the assessment differs between these two approaches.

**Phase 5: Model the Cost-effectiveness of effective SMIs**



For peer review only - http://bmjopen.bmj.com/site/about/guidelines.xhtml

For the cost-effectiveness analysis, data on short term effects on intermediate outcomes (e.g. BMI, HbA1C) from the NMA will serve as input for simulation models that extrapolate these effects into long term health effects expressed in Quality Adjusted Life Years (QALYs). These health benefits will be combined with costs (in 2019 Euro's) to estimate the incremental cost per quality-adjusted life year gained. The development of the health economic models will follow a stepped approach First a conceptual model of the disease will be developed, informed by a review of the literature. The conceptual model will inform which statistical techniques and models to use (e.g., discrete-event models, Markov models, patient level simulation models). These decisions will be guided by good practice guidelines from pharmacoeconomic communities of medical decision making and the International Society for Pharmacoeconomics and Outcomes Research (42) available data and clinical expertise available within the consortium. In all four disease models, the natural course of each of the four study conditions will be modelled with incorporation of background events and disease-specific mortality and morbidity. Intervention costs will take into account societal, healthcare, and patient perspectives, and where possible, special attention will be given to societal costs that have historically been understudied (e.g., productivity gains and changes in caregiver burden and costs in life-years gained (37). By expressing health benefits in QALYs we will be able to produce a ranking of the most cost-effective SMIs, within and across conditions. The base cases for the models will have a societal perspective, a life-time time horizon, apply differential discounting at 4% for costs and 1.5% for health (with sensitivity analysis for equal discounting at 3%). The incremental cost-effectiveness ratio's will be evaluated against a so-called v-threshold that denotes willingness to pay for a QALY (43). The WTP in the base case is assumed to be the median WTP of €24,226,- per QALY as found in the systematic review on WTP thresholds by Ryen & Svensson (2015), with sensitivity analyses using the mean WTP threshold from that same review of € 74,159, (44) and country specific thresholds where available. Reporting will include scenario analyses to evaluate structural uncertainty as well as univariate and probabilistic sensitivity analysis to evaluate parameter uncertainty.



For peer review only - http://bmjopen.bmj.com/site/about/guidelines.xhtml

294 **Phase 6: Analyse the contextual factors that promote implementation in real life**
295 **contexts**

296 For each of the most effective SMIs identified for the four chronic conditions we will
297 perform a realist systematic review (45) to identify key determinants of success (or
298 failure), such as intervention settings (e.g., whether on a primary care level or hospital
299 level care) and mechanisms (e.g., engagement processes) that produce specific
300 outcomes. At the patient level, special attention will be paid to comorbidities, gender,
301 and socioeconomic dimensions such as health literacy to better understand how these
302 influence the implementation of the selected SMIs. We will then use a modified Delphi
303 method, with experts on self-management and/or implementation of healthcare
304 interventions, to establish the importance of these contextual factors in the target
305 countries (Belgium, Germany, Greece, the Netherlands & Spain,). Experts will be asked
306 to rate the magnitude of the influence of a contextual factor of a list developed by
307 researchers. The final list of the contextual factors will be produced in a final expert
308 discussion.

309 **Phase 7: Development and piloting of the COMPAR-EU information technology platform, and**
310 **preparation for future implementation**

311 We will develop an online platform that will integrate all the information and evidence
312 synthesised during the different phases of the project. The aim is that the resources
313 included facilitate decision making for target end users (patients, healthcare
314 professionals, policymakers, researchers, and small and medium-sized enterprises
315 (SMEs)). The platform will include the following GRADE-based tools:

316 - Evidence profiles and Interactive Summary of Findings (iSoF) tables(46): these
317 presentations will provide information in different formats about the quality of
318 evidence, and magnitude of relative and absolute effects for each of the core
319 outcomes identified.
320 - Evidence to Decision frameworks (EtD): using semi-automatic templates,
321 interactive EtD frameworks (47) will be completed for a number of priority
322 questions that will take into account the magnitude of desirable and
323 undesirable effects, stakeholder views on the importance of different
324 outcomes, information on resource use and cost-effectiveness, impact on



For peer review only - http://bmjopen.bmj.com/site/about/guidelines.xhtml

equity, and other aspects like acceptability or feasibility of the interventions. The frameworks will include draft recommendations that could be then applied or adapted to different settings.

- Patient decision aids: will be developed in plain-language for all selected situations identified in the previous phases of the study. The aids will be produced in six languages (English, French, German, Spanish, Dutch, and Greek) and will include information obtained in some of the phases included in the project.

- *Development of COMPAR-EU online platform*

The COMPAR-EU online platform will feature structured interfaces tailored to the needs of different end users. It will be designed so that after answering a few simple questions, users will be guided to the tool or product that best suits their needs (Figure 2 illustrates the main products to be integrated into the platform and expected end-users).

*Figure 2. COMPAR-EU Platform, decision-making tools and other end products*

- *Piloting and refinement of decision-making tools with different stakeholders*

The COMPAR-EU platform will be piloted to gauge potential barriers to its implementation and understand how to position the decision-making tools in the target healthcare markets. Decision aids will be piloted among patients and health professionals and EtD frameworks and other products will be piloted with other stakeholders (e.g., policymakers, industry, and representatives of patient associations) that may be involved in implementing the tools in the five participating countries. Piloting will be organised in focus groups and user experience tests in simulated settings using techniques such as thinking-out-loud. Pilots with patient associations and clinicians will focus on the actual use of the decision aids, where pilots with industry and policy makers will focus on the potential reimbursement and integration into existing IT tools.

Results of the piloting will be used to further improve the tools.



For peer review only - http://bmjopen.bmj.com/site/about/guidelines.xhtml

- *Preparation of future implementation*

Even with well-developed and user-friendly decision-tools, integration in existing policy and regulatory frameworks is crucial for successful uptake of our recommendations. Using expert networks, we will assess the grey literature, current guidelines and legislation to identify and analyse relevant policy and regulatory frameworks and standards on patient participation, health technology assessment agencies, eHealth/mHealth and other topics that might influence the uptake of the self-management and decision-making tools designed within COMPAR-EU. The purpose is the ensure that current legislative, regulatory and reimbursement decisions at EU level are appropriately considered in exploiting the results of the COMPAR-EU project. We will also hold workshops with industry, pharma, and SMEs to identify business opportunities resulting from the research findings that could result in long-term sustainable dissemination.

## PATIENT AND PUBLIC INVOLVEMENT

Patients are a key component of the COMPAR-EU project from start to finish and their interests are represented by the European Patient Forum (EPF), which is one of the consortium partners. Together with other stakeholders, they will be involved in different aspects of the project including prioritisation of core outcomes and testing of interactive tools. A core group of partners, led by the EPF, will also be created to ensure co-production and establishment of explicit criteria to incorporate patient views into project outcomes, products, and communication interventions. Plain-language material will also be developed to support the main end products of the project.

## ETHICS AND DISSEMINATION

The project coordinator's (Avedis Donabedian Research Institute) requested the overall ethical approval for the project to our local Clinical Research Ethics Committee (CEIC) (the University Institute for Primary Care Research - IDIAP Jordi Gol). Ethical approval was granted on March 2018. Results are of interest to several stakeholder groups (patients, professionals, managers, policymakers and industry) and will be



For peer review only - http://bmjopen.bmj.com/site/about/guidelines.xhtml

For peer review only

disseminated in a tailored multi-pronged approach, including the creation of an interactive platform. The data generated by the project will be managed following the Golden Open access as defined by the European Commission for Horizon 2020 research projects (48).

DATA SHARING STATEMENT

COMPAR-EU project will make all its anonymized data available upon reasonable request; where required this will be at aggregated level. The main data dictionaries and databases generated by the project will be available upon requests for uses related to research and quality improvement, and potentially for commercial exploitation, subject to approval by the consortium. Data availability and access is governed by the COMPAR-EU Data Management Plan which is aligned with the EU Open Data Initiative and the FAIR Principles(49). Further details and information on how to access the data will be available from COMPAR-EU's project website (https://self-management.eu/).



For peer review only - http://bmjopen.bmj.com/site/about/guidelines.xhtml

## LIST OF ABBREVIATIONS

COS – Core Outcome Sets

EPF – European Patient Forum

NMA – Network meta-analysis

SMIs – Self-management interventions

RCT – Randomised Control Trial

For peer review only



For peer review only - http://bmjopen.bmj.com/site/about/guidelines.xhtml

## DECLARATIONS

### PATIENT AND PUBLIC INVOLVEMENT

European Patients Forum (EPF) a key umbrella organization in patient representation and European level is a partner of this project and as such has contributed to the project from its inception and design.

Furthermore we have planned for patient participation in key stages of the project, including the selection of outcomes for the Core Outcome Sets, advise towards patient end products and participating in the design and piloting of the COMPAR-EU platform and related decision making tools.

### FIGURE CAPTIONS

*Figure 1. COMPAR-EU Phases and main tasks,* presents a visual summary of the main phases of the project as described in this protocol.

*Figure 2. COMPAR-EU Platform,* decision-making tools and other end products, presents a visual summary of the projects' foreseen main products and how they relate to the key stakeholders.

### AUTHORS' CONTRIBUTIONS

- Marta Ballester contributed to the design of the project and prepared the manuscript of the paper.
- Carola Orrego, Monique Heymans, Pablo Alonso-Coello, Matthijs Versteegh, Dimitris Mavridis, Oliver Groene, Kaisa Immonen, Cordula Wagner, Carlos



For peer review only - http://bmjopen.bmj.com/site/about/guidelines.xhtml

Canelo and Rosa Suñol contributed to the design of the project and reviewed and contributed to the manuscript of the paper.

**Acknowledgments relating to this study**

The COMPAR-EU group:

Ana Isabel González (Fundación Avedis Donabedian, Avedis Donabedian Research Institute (FAD) and REDISSEC), Aretj-Angeliki Veroniki (University of Ioannina, Department of Primary Education), Claudia Valli (Iberoamerican Cochrane Centre – Biomedical Research Institute Sant Pau (IIIB Sant Pau))Claudio Alfonso Rocha Calderón (Iberoamerican Cochrane Centre – Biomedical Research Institute Sant Pau (IIIB Sant Pau)),Ena Niño de Guzmán (Iberoamerican Cochrane Centre – Biomedical Research Institute Sant Pau (IIIB Sant Pau)),Estela Camus ((Fundación Avedis Donabedian, Avedis Donabedian Research Institute (FAD)), Giorgos Seitidis (University of Ioannina, Department of Primary Education), Hector Pardo-Hernandez (Iberoamerican Cochrane Centre – Biomedical Research Institute Sant Pau (IIIB Sant Pau) and CIBER de Epidemiología y Salud Pública (CIBERESP)), Jany Rademakers (Netherlands Institute for Health Services Research, (NIVEL)), Jessica Beltrán (Iberoamerican Cochrane Centre – Biomedical Research Institute Sant Pau (IIIB Sant Pau)), Karla Salas (Iberoamerican Cochrane Centre – Biomedical Research Institute Sant Pau (IIIB Sant Pau)), Kevin Pacheco-Barrios (Fundación Avedis Donabedian, Avedis Donabedian Research Institute (FAD)), Lyudmil Ninov (European Patients' Forum), Maria Petropoulou (University of Ioannina, Department of Primary Education), Marieke van der Gaag (Netherlands Institute for Health Services Research, (NIVEL)), Montserrat León (Iberoamerican Cochrane Centre – Biomedical Research Institute Sant Pau (IIIB Sant Pau)), Nina Adrion (OptiMedis AG), Rune Poortvliet (Netherlands Institute for Health Services Research, (NIVEL)), Stella Zevgiti (University of Ioannina, Department of Primary

459 Education), Valentina Strammiello (European Patients' Forum) for their valuable assistance in conducting this proposal.

- Funding: This work was supported by European Union's Horizon 2020 research and innovation programme under grant agreement No 754936
- Competing interest statement: no competing interests.

For peer review only

## REFERENCES


1. Chang AY, Skirbekk VF, Tyrovolas S, Kassebaum NJ, Dieleman JL. Measuring population ageing: an analysis of the Global Burden of Disease Study 2017. Lancet Public Heal [Internet]. 2019 Mar 1 [cited 2019 Aug 1];4(3):e159–67. Available from: http://www.ncbi.nlm.nih.gov/pubmed/30851869

2. Patient empowerment--who empowers whom? Lancet (London, England) [Internet]. 2012 May 5 [cited 2016 Oct 4];379(9827):1677. Available from: http://www.ncbi.nlm.nih.gov/pubmed/22559881

3. Anderson G, Hopkins J. Responding to the Growing Cost and Prevalence of People With Multiple Chronic Conditions. 2007.

4. Bycroft JJ, Tracey J. Self-management support: Awin-win solution for the 21st century. N Z Fam Physician [Internet]. 2006 [cited 2017 Apr 6];33(4):243–8. Available from: http://koawatea.co.nz/wp-content/uploads/2014/10/Self-Management-support-A-win-win-solution-Bycroft-J-Tracey-J-2006.pdf

5. Aujoulat I, Marcolongo R, Bonadiman L, Deccache A. Reconsidering patient empowerment in chronic illness: A critique of models of self-efficacy and bodily control. Soc Sci Med [Internet]. 2008 Mar [cited 2017 Apr 6];66(5):1228–39. Available from: http://www.ncbi.nlm.nih.gov/pubmed/18155338

6. European Commission. Pilot project on the promotion of self-care in chronic diseases in the European Union [Internet]. [cited 2017 Apr 7]. Available from: http://ec.europa.eu/dgs/health_food-safety/funding/docs/call_sante_2015-d2-021-tech_specs_en.pdf

7. Bandura A. Toward a unifying theory of behavioral change. Psychol Rev [Internet]. 1977;84(2):191–215. Available from: http://www.apa.org/pubs/journals/rev/

8. Department of Health. The Expert Patient: A new approach to chronic disease management for the 21th century. London; 2011.




For peer review only - http://bmjopen.bmj.com/site/about/guidelines.xhtml

9. Pigott T, Shepperd S. Identifying, documenting and examining heterogeneity in systematic reviews of complex interventions. J Clin Epidemiol. 2013;66:1244 – 1250.

10. Umpierre D, Kramer CK, Leita CB, Gross JL, Ribeiro JP, Schaan BD. Physical Activity Advice Only or Structured exercise training and association With HbA 1c Levels in Type 2 Diabetes. JAMA J Am Med Assoc. 2011;305:1790–9.

11. Armstrong MJ, Mottershead TA, Ronksley PE, Sigal RJ, Campbell TS, Hemmelgarn BR. Motivational interviewing to improve weight loss in overweight and/or obese patients: A systematic review and meta-analysis of randomized controlled trials. Obes Rev. 2011;12(9):709–23.

12. Silveira JAC da, Taddei JA de AC, Guerra PH, Nobre MRC. The effect of participation in school-based nutrition education interventions on body mass index: A meta-analysis of randomized controlled community trials. Prev Med (Baltim). 2013;56(3–4):237–43.

13. McLean S, Nurmatov U. Telehealthcare for chronic obstructive pulmonary disease. Cochrane Libr. 2011;(8).

14. Inglis Sally C, Clark Robyn A, Dierckx R, Prieto-Merino D, Cleland John GF. Structured telephone support or non-invasive telemonitoring for patients with heart failure. Cochrane Database Syst Rev. 2015;(10).

15. Steinsbekk A, Rygg LØ, Lisulo M, Rise MB, Fretheim A. Group based diabetes self-management education compared to routine treatment for people with type 2 diabetes mellitus. A systematic review with meta-analysis. BMC Health Serv Res. 2012 Jan;12(1):213.

16. de Jongh T, Gurol-Urganci I, Vodopivec-Jamsek V, Car J, Atun R. Mobile phone messaging for facilitating self-management of long-term illnesses. Cochrane database Syst Rev. 2012;12(12):CD007459.

17. Tricco AC, Ivers NM, Grimshaw JM, Moher D, Turner L, Galipeau J, et al. Effectiveness of quality improvement strategies on the management of

For peer review only



For peer review only - http://bmjopen.bmj.com/site/about/guidelines.xhtml

diabetes: A systematic review and meta-analysis. Lancet. 2012;379(9833):2252–61.

18. Caldwell DM, Ades a E, Higgins JPT. Simultaneous comparison of multiple treatments: combining direct and indirect evidence. BMJ Br Med J. 2005;331(7521):897–900.

19. Salanti G. Indirect and mixed-treatment comparison, network, or multiple-treatments meta-analysis: many names, many benefits, many concerns for the next generation evidence synthesis tool. Res Synth Methods [Internet]. 2012;3(March):80–97. Available from: http://doi.wiley.com/10.1002/jrsm.1037

20. Mavridis D, Giannatsi M, Cipriani A, Salanti G. A primer on network meta-analysis with emphasis on mental health. Evid Based Ment Health [Internet]. 2015 May [cited 2017 Feb 22];18(2):40–6. Available from: http://www.ncbi.nlm.nih.gov/pubmed/25908686

21. Efthimiou O, Debray TPA, van Valkenhoef G, Trelle S, Panayidou K, Moons KGM, et al. GetReal in network meta-analysis: a review of the methodology. Res Synth Methods. 2016;7(3):236–63.

22. Dias S, Welton NJ, Sutton AJ, Ades AE. Evidence Synthesis for Decision Making 3: Heterogeneity—Subgroups, MetaRegression, Bias, and Bias-Adjustment. Med Decis Mak [Internet]. 2013;33(5):597–606. Available from: http://www.pubmedcentral.nih.gov/articlerender.fcgi?artid=3704205&tool=pmcentrez&rendertype=abstract

23. Petropoulou M, Nikolakopoulou A, Veroniki A-A, Rios P, Vafaei A, Zarin W, et al. Bibliographic study showed improving statistical methodology of network meta-analyses published between 1999 and 2015. J Clin Epidemiol [Internet]. 2016; Available from: http://dx.doi.org/10.1016/j.jclinepi.2016.11.002

24. Petticrew M, Rehfuess E, Noyes J et al. Synthesizing evidence on complex interventions: how meta-analytical, qualitative, and mixed-method approaches can contribute. J Clin Epidemiol. 2013;66:1230–43.

For peer review only



For peer review only - http://bmjopen.bmj.com/site/about/guidelines.xhtml

548 25. Reichert AR. Obesity, Weight Loss, and Employment Prospects: Evidence from a
549 Randomized Trial. J Hum Resour. 2015;50(3):759–810.

550 26. Hoogendoorn M, van Wetering CR, Schols AM, Rutten-van Mölken MPMH. Is
551 INTERdisciplinary COMmunity-based COPD management (INTERCOM) cost-
552 effective? Eur Respir J. 2009;35(1).

553 27. Boland MR, Tsiachristas A, Kruis AL, Chavannes NH, Rutten-van Mölken MP. The
554 health economic impact of disease management programs for COPD: a
555 systematic literature review and meta-analysis. BMC Pulm Med. 2013
556 Dec;13(1):40.

557 28. Tsiachristas A, Burgers L, Rutten-van Mölken MPMH. Cost-Effectiveness of
558 Disease Management Programs for Cardiovascular Risk and COPD in The
559 Netherlands. Value Heal. 2015;18(8):977–86.

560 29. Bradley EH, Curry LA, Devers KJ. Qualitative data analysis for health services
561 research: Developing taxonomy, themes, and theory. Health Serv Res.
562 2007;42(4):1758–72.

563 30. PRO-STEP Consortium. PRO STEP Project [Internet]. Available from:
564 http://ec.europa.eu/dgs/health_food-safety/funding/call_sante_2015-d2-
565 021_en.htm

566 31. EMPATHiE Consortium. Final summary report: EMPATHiE, Empowering patients
567 in the management of chronic diseases [Internet]. 2014. Available from:
568 http://ec.europa.eu/health//sites/health/files/patient_safety/docs/empathie_fr
569 ep_en.pdf

570 32. Kirwan JR, Minnock P, Adebajo A, Bresnihan B, Choy E, De Wit M, et al. Patient
571 perspective: Fatigue as a recommended patient centered outcome measure in
572 rheumatoid arthritis. In: Journal of Rheumatology. 2007. p. 1174–7.

573 33. Higgins JP, Green S. Cochrane Handbook for Systematic Reviews of [Internet].
574 Vol. Version 5., The Cochrane Collaboration. 2008. Version 5.1.0. Available from:
575 http://onlinelibrary.wiley.com/doi/10.1002/9780470712184.fmatter/summary




For peer review only - http://bmjopen.bmj.com/site/about/guidelines.xhtml

34. Chaimani A, Caldwell DM, Li T, Higgins JPT, Salanti G. Additional considerations are required when preparing a protocol for a systematic review with multiple interventions. J Clin Epidemiol [Internet]. 2017;83:65–74. Available from: http://dx.doi.org/10.1016/j.jclinepi.2016.11.015

35. Caldwell DM, Welton NJ. Approaches for synthesising complex mental health interventions in meta-analysis. Evid Based Ment Health [Internet]. 2016 Feb 1 [cited 2019 Mar 8];19(1):16–21. Available from: http://www.ncbi.nlm.nih.gov/pubmed/26792834

36. Freeman SC, Scott NW, Powell R, Johnston M, Sutton AJ, Cooper NJ. Component network meta-analysis identifies the most effective components of psychological preparation for adults undergoing surgery under general anesthesia. J Clin Epidemiol [Internet]. 2018 Jun 1 [cited 2019 Mar 8];98:105–16. Available from: https://www.sciencedirect.com/science/article/pii/S089543561731137X

37. Welton NJ, Caldwell DM, Adamopoulos E, Vedhara K. Mixed Treatment Comparison Meta-Analysis of Complex Interventions: Psychological Interventions in Coronary Heart Disease. Am J Epidemiol [Internet]. 2009 May 1 [cited 2019 Mar 8];169(9):1158–65. Available from: http://www.ncbi.nlm.nih.gov/pubmed/19258485

38. Tonin FS, Rotta I, Mendes AM, Pontarolo R, Tonin FS, Rotta I, et al. Network meta-analysis: a technique to gather evidence from direct and indirect comparisons. Pharm Pract (Granada) [Internet]. 2017 Mar 31 [cited 2019 Aug 2];15(1):943–943. Available from: http://www.pharmacypractice.org/journal/index.php/pp/article/view/943/490

39. Yepes-Nuñez JJ, Li S-A, Guyatt G, Jack SM, Brozek JL, Beyene J, et al. Development of the summary of findings table for network meta-analysis. J Clin Epidemiol [Internet]. 2019 Nov 2 [cited 2019 Oct 1];115:1–13. Available from: http://www.ncbi.nlm.nih.gov/pubmed/31055177

40. Brignardello-Petersen R, Bonner A, Alexander PE, Siemieniuk RA, Furukawa TA,

For peer review only



For peer review only - http://bmjopen.bmj.com/site/about/guidelines.xhtml

Rochwerg B, et al. Advances in the GRADE approach to rate the certainty in estimates from a network meta-analysis. J Clin Epidemiol [Internet]. 2018 Jan [cited 2019 Oct 1];93:36–44. Available from: http://www.ncbi.nlm.nih.gov/pubmed/29051107

41. Guyatt GH, Oxman AD, Schünemann HJ, Tugwell P, Knottnerus A. GRADE guidelines: A new series of articles in the Journal of Clinical Epidemiology. J Clin Epidemiol [Internet]. 2011 Apr [cited 2019 Oct 1];64(4):380–2. Available from: http://www.ncbi.nlm.nih.gov/pubmed/21185693

42. Cummins BGM. The International Society for Pharmacoeconomics and Outcomes Research. Value Heal. 2013;16(1):i.

43. Brouwer W, van Baal P, van Exel J, Versteegh M. When is it too expensive? Cost-effectiveness thresholds and health care decision-making. Vol. 20, European Journal of Health Economics. Springer Verlag; 2019. p. 175–80.

44. Ryen L, Svensson M. The Willingness to Pay for a Quality Adjusted Life Year: A Review of the Empirical Literature. Health Econ [Internet]. 2015 Oct [cited 2019 Nov 25];24(10):1289–301. Available from: http://doi.wiley.com/10.1002/hec.3085

45. Pawson R, Greenhalgh T, Harvey G, Al E. Realist review- a new method of systematic review designed for complex policies interventions. J Heal Serv Res Policy. 2005;(10):21–34.

46. Guyatt G, Oxman AD, Akl EA, Kunz R, Vist G, Brozek J, et al. GRADE guidelines: 1. Introduction—GRADE evidence profiles and summary of findings tables. J Clin Epidemiol [Internet]. 2011 Apr [cited 2019 Oct 1];64(4):383–94. Available from: http://www.ncbi.nlm.nih.gov/pubmed/21195583

47. Alonso-Coello P, Schünemann HJ, Moberg J, Brignardello-Petersen R, Akl EA, Davoli M, et al. GRADE Evidence to Decision (EtD) frameworks: a systematic and transparent approach to making well informed healthcare choices. 1: Introduction. BMJ [Internet]. 2016 Jun 28 [cited 2019 Oct 1];353:i2016. Available from: https://www.bmj.com/content/353/bmj.i2016



For peer review only - http://bmjopen.bmj.com/site/about/guidelines.xhtml

48. Guidelines to the Rules on Open Access to Scientific Publications and Open Access to Research Data in Horizon 2020 [Internet]. 2017. Available from: https://ec.europa.eu/research/participants/data/ref/h2020/grants_manual/hi/oa_pilot/h2020-hi-oa-pilot-guide_en.pdf

49. Wilkinson MD, Dumontier M, Aalbersberg IjJ, Appleton G, Axton M, Baak A, et al. Comment: The FAIR Guiding Principles for scientific data management and stewardship. Sci Data. 2016;

For peer review only



For peer review only - http://bmjopen.bmj.com/site/about/guidelines.xhtml

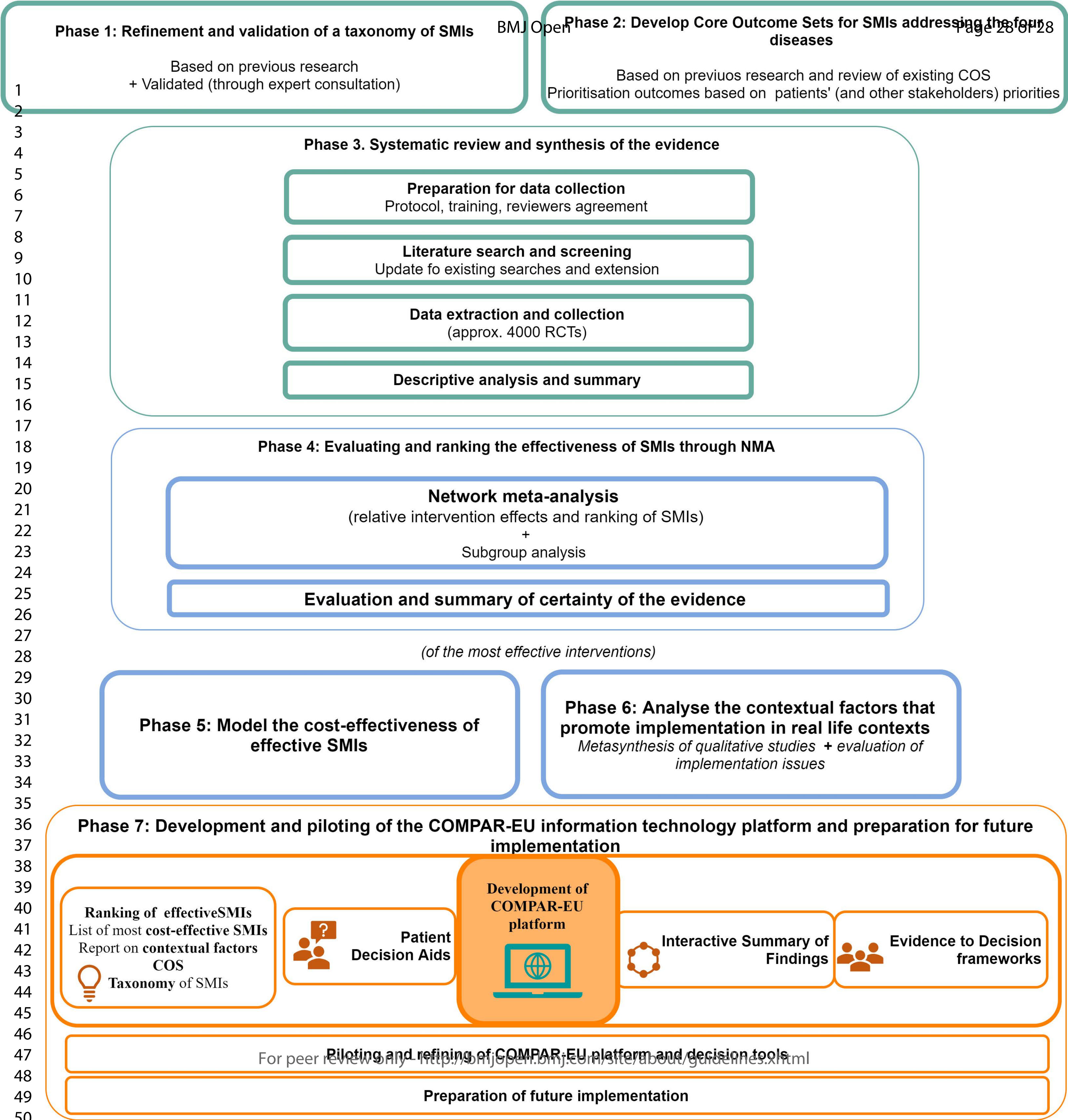


For peer review only - http://bmjopen.bmj.com/site/about/guidelines.xhtml

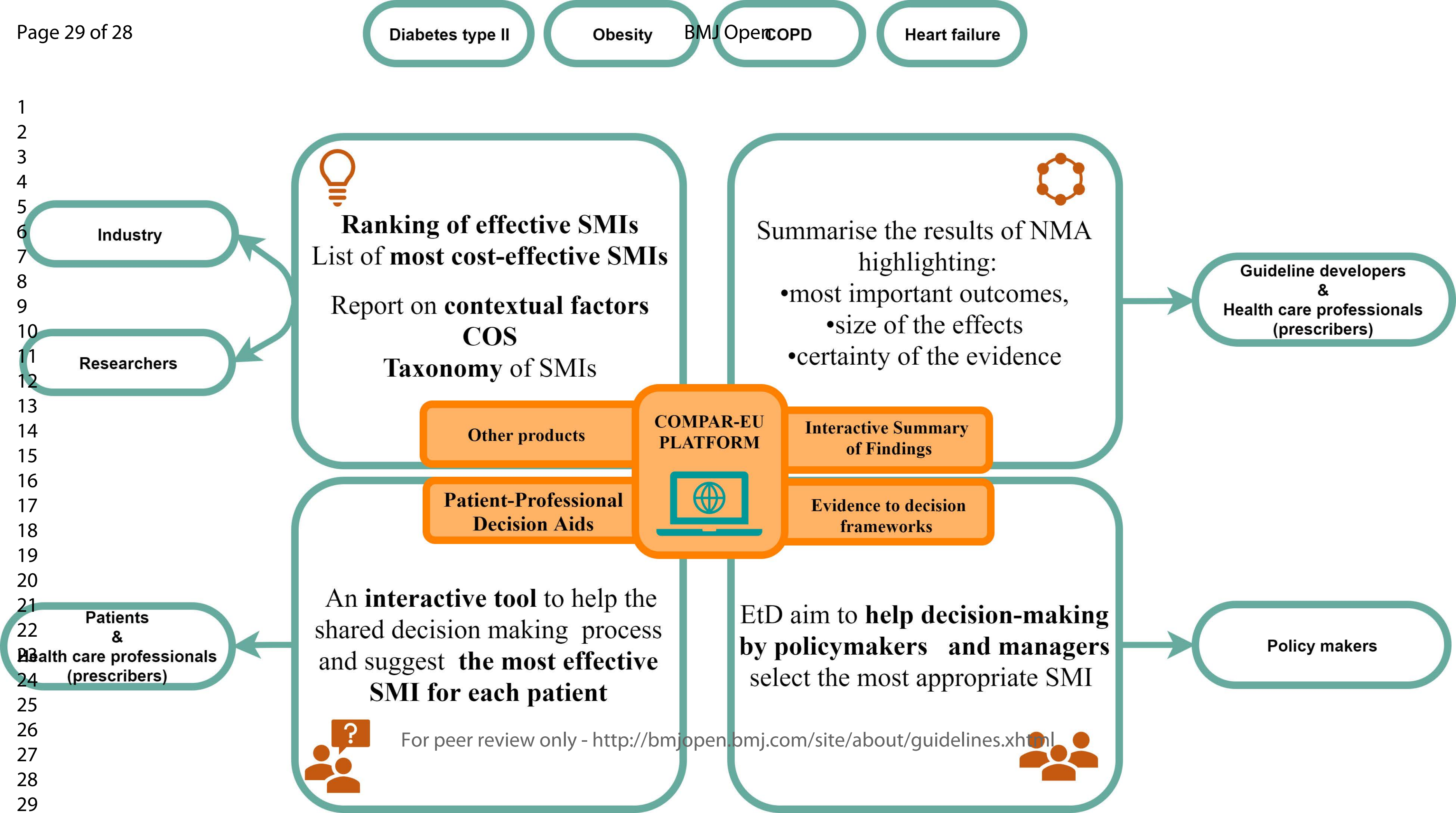
Diabetes type II
Obesity
COPD
Heart failure
Industry
Researchers
Ranking of effective SMIs
List of most cost-effective SMIs
Report on contextual factors
COS
Taxonomy of SMIs
Summarise the results of NMA highlighting:
•most important outcomes,
•size of the effects
•certainty of the evidence
Guideline developers & Health care professionals (prescribers)
Other products
COMPAR-EU PLATFORM
Interactive Summary of Findings
Patient-Professional Decision Aids
Evidence to decision frameworks
Patients & Health care professionals (prescribers)
An interactive tool to help the shared decision making process and suggest the most effective SMI for each patient
EtD aim to help decision-making by policymakers and managers select the most appropriate SMI
Policy makers
